# Impact of cold atmospheric pressure argon plasma on antibiotic sensitivity of methicillin-resistant Staphylococcus aureus strains in vitro

**DOI:** 10.3205/dgkh000277

**Published:** 2016-08-30

**Authors:** Anne Lührmann, Rutger Matthes, Axel Kramer

**Affiliations:** 1 Institute for Hygiene and Environmental Medicine, University Medicine Greifswald, Greifswald, Germany; 2 Unit of Periodontology, Dental School, University of Greifswald, Greifswald, Germany

**Keywords:** antibiotics, cold atmospheric pressure argon plasma, tissue tolerable plasma, Staphylococcus aureus, sensitivity

## Abstract

**Aim:** The antimicrobial activity of cold atmospheric pressure plasma (CAP), also called tissue tolerable plasma (TTP), could be a promising option to eradicate methicillin-sensitive as well as methicillin-resistant *Staphylococcus aureus* strains, which often colonize chronic wounds. Currently, the influence of CAP on the susceptibility of *S. aureus* to antibiotics is scarcely known, but could be important for treatment of wounds. Therefore, the aim of this study was to investigate whether CAP has an impact on the susceptibility of different *S. aureus* strains to different antibiotics.

**Method:** For assessment, the agar diffusion test with different antibiotic test disks (cefuroxime, gentamicin, oxacillin, vancomycin, ciprofloxacin, co-trimoxazole, clindamycin, erythromycin) was used. Test strains were spread on agar plates and CAP treated before the antibiotic disks were placed. After 24 hours cultivation, the inhibited growth zones were measured and differences statistically evaluated.

**Results:** In most cases, CAP had a negligible influence on the susceptibility to antibiotics. For two strains, the susceptibility significantly decreased to β-lactam antibiotics.

**Conclusion:** Because CAP can influence the antibiotic susceptibility of *S. aureus*, before conducting combined treatment with local plasma application on wounds and systemic antibiotics, their interaction must be analysed *in vitro* to exclude unwanted combination effects.

## Zusammenfassung

**Zielsetzung:** Die antimikrobielle Wirksamkeit von kaltem Atmosphärendruckplasma (CAP), auch als gewebeverträgliches Plasma (TTP) bezeichnet, könnte eine aussichtsreiche Option zur Eradikation von Methicillin-empfindlichen ebenso wie von Methicillin-resistenten *Staphylococcus aureus*-Stämmen sein, die oft chronische Wunden kolonisieren. Bisher wurde der Einfluss von CAP auf die Antibiotikaempfindlichkeit von *S. aureus* kaum untersucht. Da eine Veränderung der Antibiotikaempfindlichkeit für die Wundbehandlung relevant sein könnte, sollte der Einfluss von CAP auf die Empfindlichkeit verschiedener *S. aureus*-Stämme gegen unterschiedliche Antibiotika untersucht werden.

**Methode:** Im Agardiffusionstest wurden Antibiotikatestplättchen mit Cefuroxim, Gentamicin, Oxacillin, Vancomycin, Ciprofloxacin, Co-Trimoxazol, Clindamycin und Erythromycin eingesetzt. Die Teststämme wurden auf Agar ausplattiert und mit CAP exponiert, bevor die Testplättchen aufgelegt wurden. Nach 24 h Bebrütung wurden die Inhibitionszonen gemessen und statistisch auf Unterschiede geprüft.

**Ergebnisse:** In den meisten Fällen war die Einfluss von CAP auf die Antibiotikaempfindlichkeit zu vernachlässigen. Für zwei Stämme wurde die Empfindlichkeit gegenüber β-Lactam-Antibiotika signifikant herabgesetzt.

**Schlussfolgerung:** Da CAP die Antibiotikaempfindlichkeit beeinflussen kann, sollten vor beabsichtigter kombinierter lokaler CAP-Behandlung und gleichzeitiger systemischer Antibiotikagabe Interaktionen *in vitro* untersucht werden, um unerwünschte Kombinationseffekte auszuschließen.

## Introduction

Increasingly, healthcare-associated infections are caused by drug-resistant microbial pathogens [1]. Of special interest are methicillin-resistant strains of *Staphylococcus aureus* (MRSA), which have a high incidence rate [2] and are mainly associated with infections on implants or catheters [3] but also on wounds [4]. The emergence and spread of MRSA in hospitals poses a challenge for infection control interventions and limits treatment options [5]. Therefore, new treatment strategies against antibiotic resistance are important.

Cold physical plasma has been the focus of increasing interest as new option in medicine, especially for wound treatment [6] and cancer therapy [7]. Plasma generated at atmospheric pressure within the physiological temperature range can be suitable for skin and wound treatment; therefore, different plasma devices have been developed and used successfully [8], [9]. In wound treatment, plasma can promote the wound healing process [10] and is microbicidally active against a wide spectrum of microbes [11], [12], [13], including multidrug-resistant strains [14] as well as microorganisms embedded in biofilms [15], [16]. Pharmacological effects, including microbicidal efficacy, are mainly caused by reactive oxygen and nitrogen species already present in the plasma flow or generated by contact of the plasma with liquids or organic substances [8].

An extensively investigated plasma source is the kinpen09^®^ (neoplas GmbH, Greifswald, Germany) [17], which generate a tissue tolerable plasma (TTP) with defined parameters [6], [18]. TTP seems to be a hopeful new therapeutic option, for instance for chronic wound treatment or to treat MRSA carriers [6].

The antimicrobial efficacy of plasma against multidrug-resistant pathogens, including MRSA, on agar plates and embedded in biofilms have been investigated by different authors [14], [19], [20] and showed different strong effects between drug-sensitive and drug-resistant *Staphylococcus aureus* strains [19], [21], [22]. However, how plasma treatment of microorganisms affects antibiotic resistance of the same is currently unknown. Maybe, plasma could reduce or increase the resistance of drug-resistant strains. Therefore, it was of interest as a pilot study to evaluate the influence of TTP application on the resistance of eight genetically different *Staphylococcus (S.) aureus* strains to different antibiotics using a disk-diffusion technique. The genetic differences of the selected strains were distinguished in carrying the *mecA* and *luk-P* genes. The gene *mecA*, encode the penicillin binding protein 2A, a protein that has an affinity to adhere to beta-lactam antibiotics and reduce hereby their biological effects, and is a characteristic part of the genome of MRSA strains. The gene *luk-P*, an important virulence factor, because it can express the membrane toxin Panton-Valentine leukocidin (PVL). The presence of *luk-P* could influence the activity of other vital relevance genes affected by cell stress. Bacterial cell stress can be caused by CAP [23] or antibiotics [24].

## Material and methods

### Test organisms

The genetic backgrounds of *mecA* and *luk-P* differs between the *S. aureus* strains used, so that the strains carry *mecA* or *luk-P* or both genes together in their genome. The SZ strains (SZ 148, SZ 179) were obtained from the nasal cavity of asymptomatic carriers from Sczcecin, Poland, and H strains (H 2966, H 3163, H 5391) from mature furuncles of furunculosis patients during the acute phase of skin infection, or during abscess incision by a surgeon as described in Masiuk et al. [25]. The *S. aureus* strains numbers 05-01825 and 98-00406 were received from the Robert Koch Institute, Wernigerode, Germany, and were analysed by Strommenger et al. [26]. The *S. aureus* ATCC 6538 strain served as reference strain, without genetic background of *mecA* and *luk-P*. The genetically availability of *mecA* and *luk-P* is shown for each strain in the result table (Table 1 (Tab. 1)).

### Bacterial cultivation and preparation for treatment

The *S. aureus* strains were grown on Columbia blood agar (BD, Heidelberg, Germany) for 24 h at 37°C. The bacterial lawn was removed with 3 ml of sterile 0.9% saline (NaCl) solution and an applicator. After bacterial resuspension, a cell concentration to 10^8^ colony-forming units (CFU) was adjusted by densimetric measurement and a serial dilution (10^8^, 10^7^, 10^6^ CFU/ml) was prepared in NaCl solution. The 10^8^ CFU/ml suspension was used for argon plasma and argon gas application, and the 10^8^, 10^7^ and 10^6^ CFU/ml suspensions were used to inoculate the agar plates for the control (no treatment) and the antibiotic test alone. For inoculation, 100 µl of the dilutions were spread on Oxoid Iso-Sensitest™ agar plates (Oxoid, Thermo Fisher Scientific, Wesel, Germany). A note to the chosen agar: The often used Müller-Hinton agar for antibiotic diffusion test is not suited in combination with plasma treatment. Therefore, the Iso-Sensitest agar was preferred. Before treatment, the inoculated agar plates dried for 20 min at room temperature under laminar air flow to avoid a fluid inoculum film during treatment.

Each strain and treatment type was performed 3 times: twice for plasma treated strains (n=6) and once for the controls and gas treatment (n=3). After treatment, the bacteria were incubated for 24 h at 37°C. These were later statistically analyzed. 

The different dilutions for the controls were used, because argon plasma reduced the bacterial load on the agar surface, and different cell densities of bacterial load on the agar surface will show different results in the antibiotic agar diffusion test. Thus, three subsequent dilutions were chosen for the controls to obtain results which are comparable to the plasma-treated samples. 

### Treatment with argon plasma

A radio frequency plasma pen (kinpen09^®^, neoplas GmbH, Greifswald, Germany) using argon (99.999% pure) as carrier gas served as the plasma source. The device operated with 1.1 MHz at 2–6 kVpp with a maximal input DC power of 3.5 W to the hand-held unit [27]. The gas flow was adjusted to 5 slm (standard liters/min) controlled by a mass flow controller (MKS Instruments, Munich, Germany). During plasma generation open to room air, the spatial afterglow plasma plume was 7 mm long. The plasma effect on the surface reaches a diameter of 3 mm around the centre of the plasma plume, which was evaluated before.

**Figure 1:**
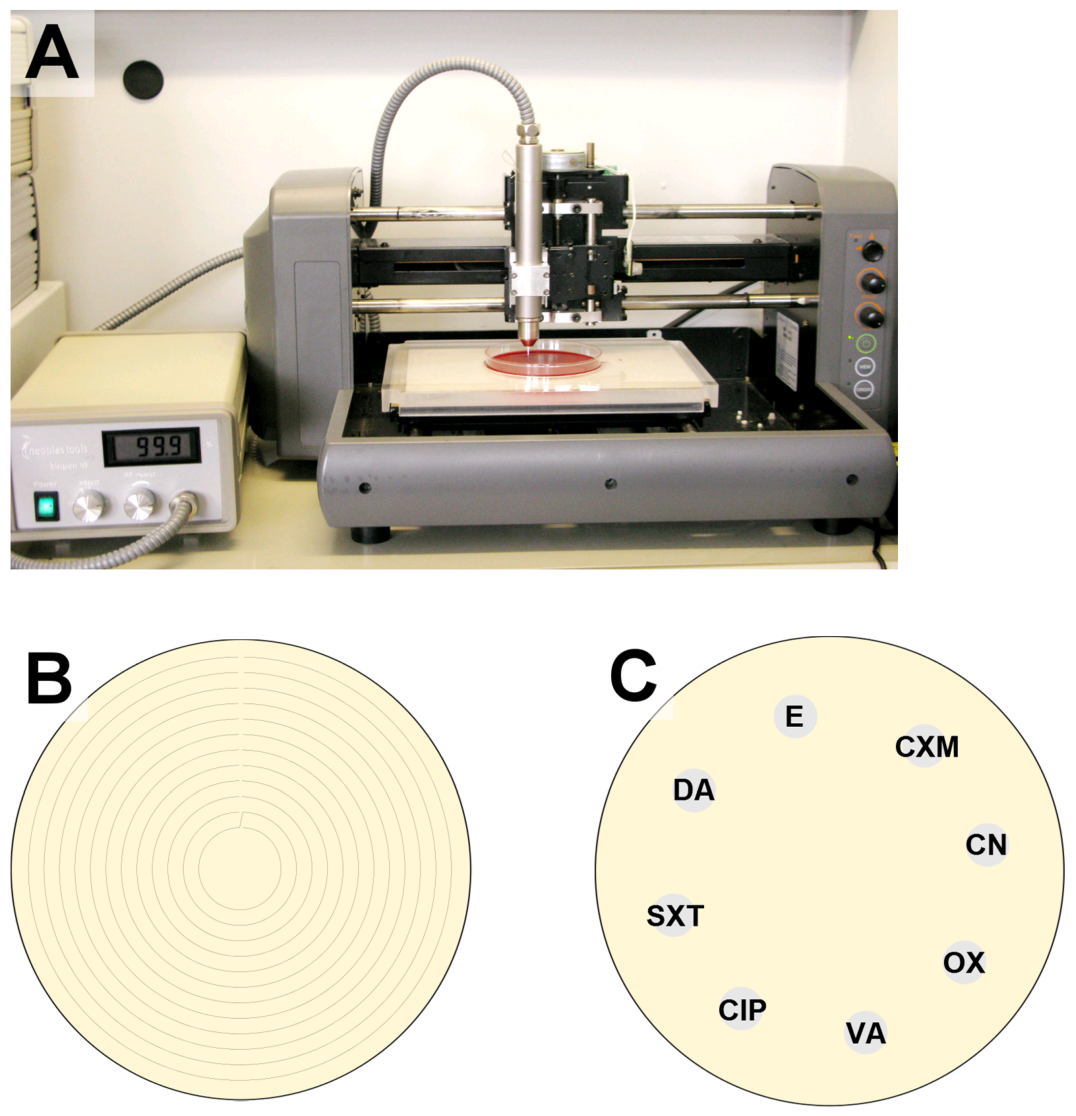
Setup of the plasma application system (A), illustration of the movement pattern for the plasma or gas application (space between the circles = 3 mm) on agar plate (B), and placement of disks in on the prepared agar plates in the antibiotic sensitivity test (CXM – cefuroxime, CN – gentamicin, OX – oxacillin, VA – vancomycin, CIP – ciprofloxacin, SXT – co-trimoxazole, DA – clindamycin, E – erythromycin) (C)

To use for treatment, the pen was fixed in a computer-controlled x/y/z stage above the prepared agar plate at a distance of 7 mm (Figure 1A (Fig. 1)) and was moved once along a spiral pattern (Figure 1B (Fig. 1)) at a speed of 10 mm/s. The distance between the resulting concentric circles was 3 mm. This resulted in a total treatment time of 6 s at all treated points on the agar plate, a short treatment time to achieve a sublethal plasma dose for the most bacterial cells.

During treatment, an increase in temperature generated on the surface is not detectable, because the plasma-surface contact time is too short by moving plasma pen. A plasma treatment on fixed place for 30 s showed a temperature of 22.6°C. After 60 s treatment, the temperature increased to 24.9°C. That corresponds to a temperature rise of 4.4°C and 6.7°C after 30 s and 60 s punctual treatment time. In order to measure the surface temperature, a non-contact method by using the infrared radiation emitted by the substrate was used [28] by using a pyrometer (digital infrared camera; Fluke Deutschland GmbH, Glottertal, Germany). 

As plasma control, the pure argon gas without plasma ignition was applied in the same manner like the plasma application. The CAP and gas were applied as a pre-treatment, before antibiotic disks were positioned, or as sole treatment, to get respective controls.

### Antibiotic susceptibility test

Within 15 min after plasma or gas treatment (time for handling), the antibiotic disks (6 mm, BBL™ Sensi-Disc™, BD, Heidelberg, Germany) for the susceptibility test were positioned on the prepared agar plates (Figure 1C (Fig. 1)) and incubated for 24 h at 37°C. Antibiotic test disks used were cefuroxime (CXM) 30 µg, gentamicin (CN) 10 µg, oxacillin (OX) 5 µg, vancomycin (VA) 30 µg, ciprofloxacin (CIP) 5 µg, co-trimoxazol (SXT) 25 µg, clindamycin (DA) 2 µg and erythromycin (E) 15 µg. All disks were stored in their containers at –20°C until used in these experiments. After 24 h of incubation at 37°C, the diameter of growth inhibition zone was measured. A strain was generally considered resistant to the given antibiotic if the growth inhibition zone had a diameter of less than 15 mm, as based on the Performance Standards for Antimicrobial Susceptibility Testing, Twenty-second Informational Supplement, 2013 [29]. A growth-inhibition-zone threshold (a set diameter) to determine bacterial resistance for each antibiotic separately was not defined, because only the differences of growth inhibition zones were of interest to decide whether the susceptibility after plasma treatment increased or decreased. Because ISO-Sensitive agar was used instead of Mueller-Hinton agar, the guidelines can only serve as orientation.

## Statistical analyses

**Figure 2:**
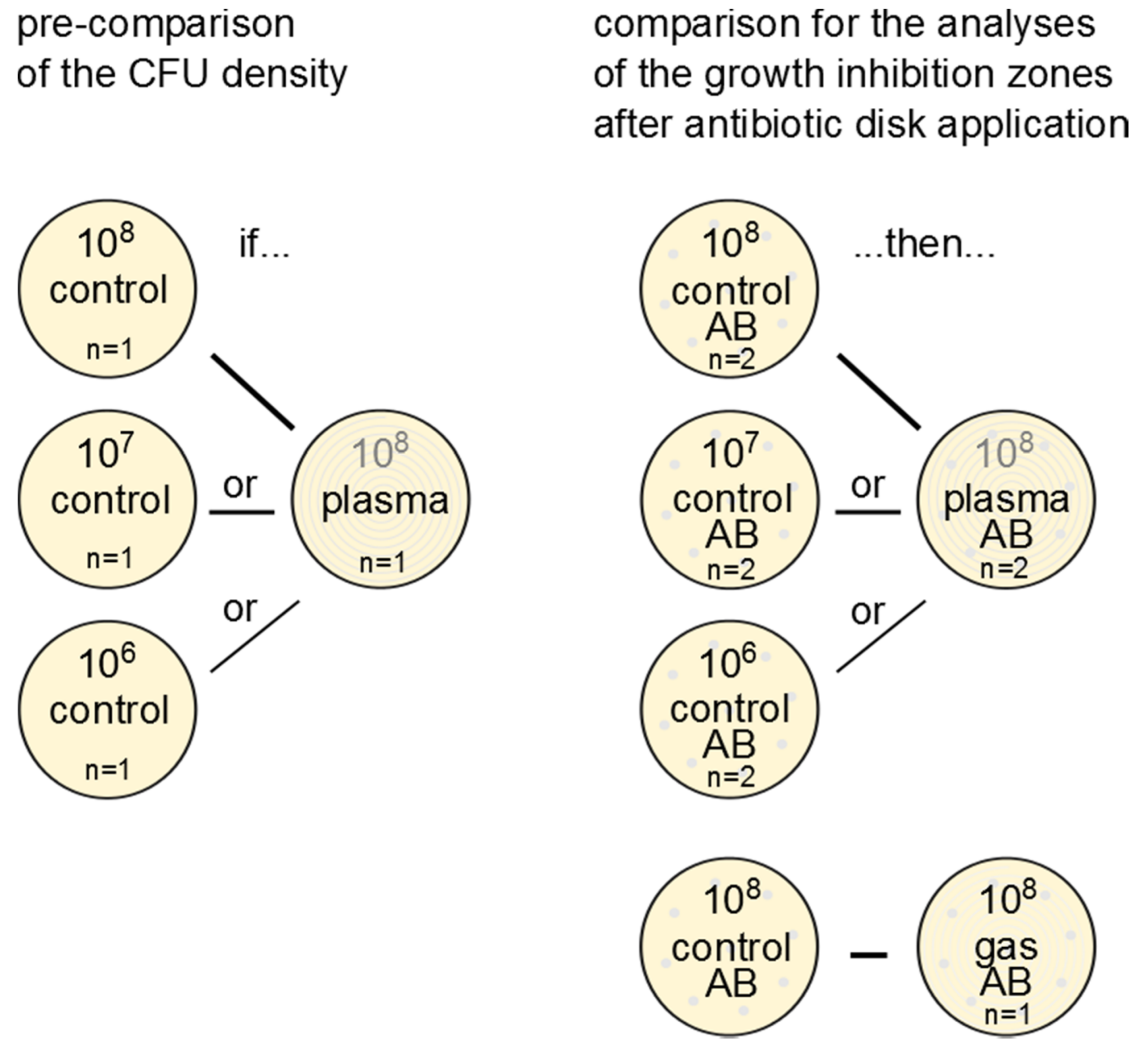
Illustration of the test scheme (untreated control, plasma and gas treatment) and choice of samples after plasma treatment for comparison of the growth inhibition zone caused by application of the antibiotic sensitivity test disks (AB)

The diameter of the growth inhibition zone was measured in mm and was used in the statistical evaluation to compare plasma treatment, gas treatment, and the control (without plasma or gas treatment) in each strain. The detection limit was a radius of 1 mm from the disk’s edge, which corresponds to a limit of diameter measurement of 8 mm. In the case of detection limit, no growth inhibition zone was observable and the value was set 6 (diameter of the antibiotic discs). The comparison of the measured antibiotic-induced growth inhibition zones of plasma-treated *S. aureus* strains to their respective antibiotic control was chosen depending on the result of the comparison of the plasma-treated agar plates to the different plated dilutions (10^8^, 10^7^ or 10^6^ CFU/ml) of the untreated control series (Figure 2 (Fig. 2)). Control plates with analogical density of CFU after plasma treatment were used for the comparisons.

The mean diameter of the growth inhibition zone in each test run of the plasma treatment, gas treatment, and the antibiotic control (without plasma or gas treatment) of each strain was used for the statistical evaluation. Statistical differences were analysed with the Mann-Whitney *U*-test followed with Bonferroni *p*-value correction for multiple comparisons using statistical analysis software (Prism, GraphPad, USA).

## Results

The changes of the growth inhibition zones produced by antibiotic disks with or without argon plasma or argon gas pre-treatment varied between the different *S. aureus* strains examined (Table 1 (Tab. 1)). Of the five strains with a genetic *mecA*-positive background, only the *S. aureus* strain 98-00406 showed resistance against the β-lactam antibiotics cefuroxime and oxacillin in the antimicrobial diffusion sensitivity disk test on agar. The *S. aureus* strain 98-00406 showed additional resistance to the aminoglycoside antibiotic gentamycin, the fluoroquinolone antibiotic ciprofloxacin, and the translation-inhibiting antibiotics clindamycin and erythromycin. Clindamycin and erythromycin resistances were also shown for the *S. aureus* strains SZ 179 and H 5391. The *S. aureus* strain 05-01825 showed less susceptibility to ciprofloxacin and erythromycin (Table 1 (Tab. 1)).

The plasma treatment influenced the growth inhibition by the antibiotic test disks in some cases. A statistically significant decrease in the growth inhibition zone of 6.3 mm with cefuroxime and of 14.5 mm with oxacillin was shown for the *S. aureus* strain SZ 148, as well as a decrease for strain 05-01825 of 36.8 mm by oxacillin (p<0.05) (Table 1 (Tab. 1)). No test organism demonstrated a significantly increased growth inhibition zone after plasma treatment.

Interestingly, argon gas treatment without plasma ignition in some cases showed a decreased growth inhibition zone (Table 1 (Tab. 1)) for co-trimoxazole – an inhibitor of the microbial tetrahydrofolate biosynthesis – which was significantly different from the growth inhibition by plasma for the strains without the genetic background of *mecA* H 2966, H 3163 and ATCC 6538. 

The existence of the gene *luk-P* in the *S. aureus* genome does not seem to influence susceptibility to antibiotics after argon plasma or argon gas treatment.

## Discussion

New methods based on cold atmospheric pressure plasma are currently being developed for the treatment of chronic wounds with additional microbicidal efficacy to inactivate drug-resistant microorganisms in the wounds. Topical application of antibiotics for wound treatment is obsolete due to the increased risk of drug resistance, but systemic use of antibiotics is indicated if septic metastasis occurs [30]. For the future of wound treatment, the combination of local plasma application with systemic antibiotic therapy is a promising option. For this to be effective, knowledge of the influence of plasma treatment on microbial sensitivity to antibiotics is an essential precondition, because plasma can influence the sensitivity of microorganisms to antibiotics. The main effect of plasma is caused by the plasma-generated reactive oxygen and nitrogen species, which interact with the cells and the surrounded media, changing the cells’ environment in terms of increased oxidation processes and decreased pH. For the used kinpen 09, in argon plasma radiating effluent, excited species of hydroxyl (OH), nitrogen (N_2_), atomic oxygen (O), and argon (Ar) can generate [31], [32], which can react to further species in contact with environmental air. A study showed that induced stress and changes of environmental pH can cause an increased resistance to antibiotics [33], [34]. Thus, an increased resistance to antibiotics and decreased growth inhibition zones of *S. aureus* strains were expected in our study.

Otherwise, if plasma could lessen drug resistance of microorganisms by increasing their sensitivity to antibiotics, this could open new treatment options against multidrug-resistant microorganisms.

*S. aureus* is one of the most problematic pathogens in nosocomial infections and can show different single or multiple resistances against antibiotic drugs. For this study, eight different *S. aureus* strains with or without a genetic background of *mecA* and or *luk-P* were used. The tests were carried out on Iso-Sensitest agar, because it is suited for the antibiotic diffusion test and showed only a minor effect on bacterial growth if agar was plasma treated, before being inoculated with microorganisms. So, the influences to an antimicrobial effect caused by the media was negligible. The recommend Mueller-Hinton agar for the antibiotic agar diffusion test showed clear antimicrobial effects after plasma treatment and was not suited therefore for the present study (determined in pre-tests).

The plasma treatment time used, approximately 6 s per treated area, was very short, considering that tissue tolerability was shown for up to 60 s of plasma exposure time [35]. Because plasma efficacy on planktonic cells on agar plates is very high, a short treatment time was necessary to maximize the number of cells remaining after plasma treatment, as the agar diffusion test requires a large number of cells. To circumvent influence of possible differences of viable bacterial cells after CAP application, the diameter of the growth inhibiting zone after antibiotics of CAP-treated bacteria was compared with the suited control plate, which showed the same bacterial colony-density like the plates after CAP treatment without antibiotics (Figure 2 (Fig. 2)). 

The results suggest that argon plasma can increase the resistance against antibiotic drugs, but the effect was mostly very low and not significant. However, in three cases, the increased resistance was significant for the effects of β-lactam antibiotics, especially regarding oxacillin for the test strains SZ 148 and 05-01825. The existence of the virulence factor *luk-P* (Panton-Valentine leukocidin) did not play a role in plasma-treated *S. aureus* growth in an antibiotic environment and thus constitutes no risk for combined therapy with plasma and antibiotics. The study of Hoon Park et al. 2015 [36] could demonstrate that an Ar plasma of a surface dielectric barrier discharged (DBD) plasma device reduced β-lactamase activity and increased therefore the susceptibility against β-lactam antibiotics. The plasma treatment time in the study of Hoon Park et al. was 300 s instead of 6 s of this study, resulting in different energy load, which can be one reason for the increased susceptibility against β-lactam antibiotic of plasma treated *S. aureus* cells. Another reason could be the gas flow, which was 1 slm in the study of Hoon Park et al. and 5 slm in this study. Because, the argon gas treatment also caused increased drug resistance, and showed significantly stronger effects than the plasma treatment, especially for the *S. aureus* strains 05-01825, H 2966 and H 3163, compared to the control or to the plasma treatment for most of the antibiotic substances examined, which was not expected. Argon is an inert gas and no effect on microorganisms is expected. However, studies on the proteomic and transcriptomic pattern of *Bacillus subtilis* after argon gas treatment showed an induced activity of genes known for stress responses [23]. Perhaps the genetic response to pure argon has a stronger influence on the antibiotic susceptibility of *S. aureus* than does the genetic response to argon plasma. Nevertheless, the reason why plasma showed a weaker effect than the pure argon is currently unknown. Possibly the mechanic influence of the gas flow of 5 slm cause the reduced susceptibility against antibiotics. If plasma increases the susceptibility against β-lactam antibiotics, like reported in Hoon Park et al. 2015 [36] by reduced β-lactamase activity, the CAP maybe reduced the gas flow effect, which could explain a lower reduction of the susceptibility against antibiotics after CAP treatment than after application of pure argon.

Additionally, pure argon treatment of *S. aureus* without subsequent antibiotic contact showed no effect (n=4 of each strain). The CAP effect without subsequent antibiotic contact can vary low between tested strains in that test setup. A previous study, with another test setup, showed also differences in correlation to the genes *mecA* and *luk-P*, which was only marginal [37]. 

In this study, a discrepancy between the PCR analyses – which detected the genetic code for *mecA* gene – and the lack of resistance to oxacillin or cefuroxime after the antibiotic susceptibility test was shown. Only strains 05-01825 and 98-00406 carrying *mecA* showed a resistance against oxacillin (strain 05-01825 only after argon application), but not strains H 5391, SZ 148, and SZ 179. Such a discrepancy between a genetic *mecA* background and lack of resistance against oxacillin in the agar-diffusion test does not seem unusual and is described in the literature [38], [39], [40].

This pilot study is one of the first investigations on the impact of argon plasma at low temperatures, operated using tissue tolerable parameters, on the susceptibility of different *S. aureus* strains to antibiotic drugs. One of the limits is that the *in vitro* study cannot reflect possible effects *in vivo*. Infections *in vivo* are mostly associated with multispecies biofilms in a very complex wound fluid milieu. However, the results show that plasma treatment can increase the resistance of *S. aureus* against antibiotics, which should be reason enough to carefully consider such effects before applying plasma to infected wounds. A decrease of bacterial drug resistance caused by plasma influence seems not possible and could be no option to support antibiotic treatment of microbially colonized wounds. Further research should test longer plasma treatment times and include different species analysed with different methods to exclude possible plasma effects caused by interaction with the media used. The impact of pure argon on the susceptibility of microorganisms to antibiotics could be a worthwhile study.

## Conclusion

For treatment of microbially infected wounds, plasma is being examined as a new potential therapy option. Our results showed that although argon plasma can influence the susceptibility of *Staphylococcus aureus* strains to antibiotics, the effect was mostly not significant. However, in two cases, a significant increase in resistance against β-lactam antibiotics was shown. This suggests that lower efficacy of antibiotics after plasma treatment should be considered if a combined treatment of plasma and antibiotics is planned. Further research to reflect more realistic treatment conditions is necessary before plasma applications on infected wounds can be recommended for combined treatment with antibiotics.

## Notes

### Acknowledgement

The authors thank Dr. Silva Holtfreter and Dr. Julia Kolata (Department of Immunology, Ernst-Moritz-Arndt-University, Greifswald) for providing of the *Staphylococcus aureus* strains.

### Funding

This study was conducted within the multi-disciplinary cooperative research program “Campus PlasmaMed”, in particular within the sub-project “PlasmaCure”, and was supported by a grant from the German Ministry of Education and Research (BMBF, grant No. 13N11181). 

### Competing interests

The authors declare that they have no competing interests.

## Figures and Tables

**Table 1:**
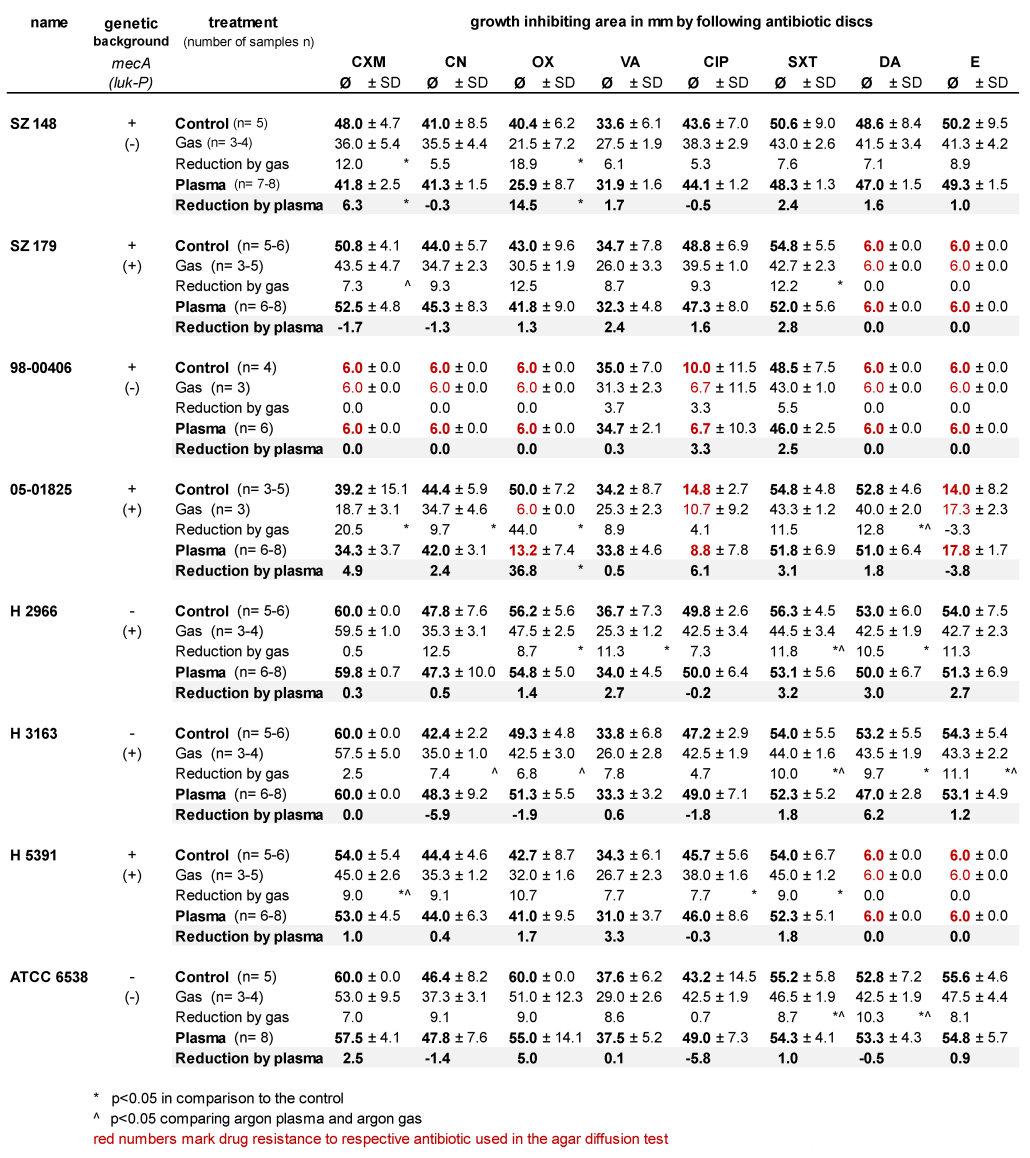
Measured growth inhibition zone of the eight investigated clinical *Staphylococcus aureus* strains under control and plasma- or gas-treatment conditions and incubated with antibiotic susceptibility test disks (CXM – cefuroxime, CN – gentamicin, OX – oxacillin, VA – vancomycin, CIP – ciprofloxacin, SXT – co-trimoxazol, DA – clindamycin, E – erythromycin) in mm (diameter), with standard deviation (SD) and number of samples (n). The reduction values show the difference from the control. Red numbers represent a growth inhibition zone less than 15 mm, which suggests resistance to the respective drug. The genetic background of *mecA* and *luk-P* of each strain is shown, the sign “+” shows that the strain carry that gene, the sign “-” shows the absence of that gene in their genome.
